# DNA/Cell Mass Homeostasis: Coordinating DNA Replication and Cell Size with Central Carbon Metabolism During Bacterial Growth

**DOI:** 10.3390/genes17060695

**Published:** 2026-06-15

**Authors:** John Herrick

**Affiliations:** Independent Researcher, 3, Rue des Jeûneurs, 75002 Paris, France; jhenryherrick@yahoo.fr

**Keywords:** DNA replication, DnaA, ribonucleotide reductase, nucleoid complexity, nucleotypic effect, DNA mass homeostasis

## Abstract

DNA/cell mass homeostasis is a pervasive feature of living organisms. As the cell grows in response to nutrient availability, it must duplicate each chromosome once and only once each division cycle. Across the eukaryote Tree of Life, cells differ in their sizes in a manner that depends directly on the amount of DNA they harbor, what has been termed the “nucleotypic effect”: cell size expands or contracts as DNA content increases or decreases. In eukaryotes, any deviation from DNA/mass homeostasis results in the deregulation of the developmental program and the initiation of carcinogenesis and other genetic pathologies. In bacteria, deviation from, or perturbation of, DNA/mass homeostasis alters important physiological features such as the cell cycle timing of DNA replication initiation and the coordination of initiation with replication termination and cell division. In prokaryotes, the timing of initiation occurs at a relatively constant and growth-rate-invariant mass, termed the initiation mass (*Mi*), and depends strictly on DNA replication fork rates and membrane biogenesis. Complex “machines”, frequently referred to as hyperstructures or factories, mediate the phase transitions that define the different periods of the bacterial cell cycle. The following will examine how DNA/mass homeostasis maintains a balance between DNA replication initiation and elongation in order to gate the phase transitions that organize the cell cycle in time and space.

## 1. Introduction

### 1.1. Coupling Between Rate of DNA Synthesis and Growth Rate

In bacteria, the rate of DNA synthesis is coupled to cell growth rate and, in particular, to the increase in cell volume [1]. This coupling occurs at the level of DNA replication initiation rather than at the level of the replication fork rate: the C-period, or duration of chromosome duplication, is growth-rate-invariant at fast growth rates and lasts approximately 40 to 44 min. The initiation process depends on the ATP-bound form of DnaA, which is inactivated by the hydrolysis of ATP to ADP [2]. ATP plays an allosteric regulatory, as opposed to a bio-energetic role, in replication initiation since DnaA unbound by ATP remains competent to initiate DNA synthesis [3].

Meselson and Stahl first observed the constancy of the C-period, stating that “the generation time is very nearly the same for all DNA molecules in the population. This raises the question of whether in any one nucleus this clock regulates nuclear and cellular division as well” [4]. Cooper and Helmstetter later confirmed Meselson–Stahl’s hypothesis and established the classical paradigm of the bacterial cell cycle [5]. Based on these observations, Cooper proposed the “Continuum Model” [6], which held that macromolecular synthesis in bacteria proceeds at an invariant rate until the cell reaches a critical mass, at which point the cell divides. Lacking a detailed mechanistic explanation, the model remains a hypothesis.

The increase in cell volume depends strictly on the growth rate of the cell and the rate of membrane biogenesis [7], while membrane growth rate (cell volume and length) depends itself directly on the rate of ATP synthesis and the availability of ATP to drive macromolecular synthesis and the accumulation of biomass. The timing of replication initiation is thus necessarily coupled to cell growth rate and consequently to a given cell mass that is approximately invariant at all growth rates: the so-called initiation mass (*Mi*). How the cell couples initiation timing to *Mi* has remained enigmatic since it was first proposed in 1968 [8]. To this day, the concept remains controversial but is nevertheless widely accepted.

### 1.2. The Interplay Between Central Carbon Metabolism and DNA Replication

Recent studies have revealed a link between Central Carbon Metabolism (CCM) and DNA replication [9,10,11,12,13,14,15,16]. The metabolic genes involved are suppressors of themo-sensitive DNA replication initiation and elongation genes, referred to as *dnaA*(ts). The effect on DNA replication was found to be direct and independent of perturbations in cell growth rate [17]. Presumably, but as yet experimentally undemonstrated, the effect of CCM mutants on DNA synthesis might be mediated by cell membrane growth rate and possibly cell size [18]. How these suppressors operate to restore DNA synthesis in *dnaA*(ts) mutants remains a question of continuing interest, but most, if not all, appear to converge, directly or indirectly, on nucleotide synthesis and flux (see below).

The former feature, an effect of cell size, is supported by the observation that perturbing replication forks (experimentally slowing them) results in increased nucleoid complexity (*NC*), according to which DNA content (number of replication points) regulates cell size [19]. Conversely, experimentally increasing fork rates results in a shortened C-period without affecting cell division [20]. Hence, replication fork rate corresponds to a major parameter governing the rate of initiation and cell size: fork rate and initiation frequency during a single cell cycle form what has been termed a “homeostatic pair” that maintains DNA/cell mass equilibrium [21]. The latter proposal is consistent with Cooper’s Continuum Model [22], which has gained increasing support.

### 1.3. Growth Rate Dependent and Independent Variation in Chromosomal Content

The bacterial cell cycle is heuristically divided into three distinct periods. The B-period (variable in duration, >0 min) corresponds to cell growth between the time of cell division and the time of initiation of DNA replication. The C-period (about 40 min) defines the duration of DNA synthesis between the time of initiation and the time of termination of chromosomal duplication. The D-period (about 20 min) corresponds to the time between termination and cell division. Since cell growth rate varies depending on nutrient availability, with a minimum doubling time of about 20 min, the C-period + D-period defines two distinct growth regimes: a fast growth regime when the doubling time is shorter than the C-period (τ < 60 min), and a slow growth regime when the doubling time is longer than the C-period (τ > 60 min).

Consequently, during fast growth, the initiation of DNA replication occurs in the mother cell before cell division. This results in chromosomes with multiple replication forks that continue synthesizing DNA during division into the two daughter cells. The number of replication forks per chromosome therefore depends on nutrient availability and growth rate and can be expressed as *n* = C/τ, where “*n*” corresponds to the number of replicating points in the cell [23,24]. Two other related quantities are the origin/terminus ratio (o/t = 2*^n^*) and the nucleoid complexity (*NC* = (2*^n^* − 1)/nln2. Conversely, the slow growth regime is characterized by the emergence of the B-period that varies in duration according to nutrient availability. Hence, the duration of the B-period determines the cell doubling time in the slow growth regime.

Variation in cell size, or volume, is a salient feature of varying growth rate. Cell size increases with growth rate while cell mass varies in direct proportion to cell size [25,26,27,28,29,30]. Cell size homeostasis is therefore maintained at all growth rates. At the same time, DNA content increases or decreases according to cell size, with larger cells containing more chromosomes (4 to 8 compared to 1 to 2). This DNA/mass ratio, or homeostasis, holds independently of growth rate: slow-growing cells experimentally enlarged 7- to 8-fold, compared to control cells growing at similar rates, have 3- to 4-fold more DNA content, and up to eight chromosomes per cell [31].

In addition to cell size homeostasis and DNA/mass homeostasis, it has been repeatedly observed that altering initiation timing results in either a longer C-period (premature initiation) or in a shorter C-period (delayed initiation), suggesting a possible homeostatic relationship between replication initiation timing and the elongation rate of the DNA chain: “there is a homeostatic mechanism that adjusts the C-period in response to alteration of the initiation time, irrespective of the mechanism by which the initiation time is altered” [28].

That the initiation activity and the elongation activity could automatically respond to each other [32]—and hence “sense” each other’s level of activity independently of the mechanism by which initiation timing is altered—suggests a deeper physiological link between cell size homeostasis, DNA/mass homeostasis and initiation–elongation homeostasis. The following will review how replication fork rates couple initiation–elongation homeostasis to cell size homeostasis and DNA/mass homeostasis, and will address the Meselson–Stahl hypothesis that the growth rate invariance of the C-period might act as a molecular clock governing not only DNA replication but also the cell cycle itself.

## 2. Is the Nucleotypic Effect Analogous to Nucleoid Complexity?

### 2.1. The Relationship Between Ploidy and Cell Size in Bacteria

In eukaryotes, it is well known that cell size varies directly in proportion to DNA content: cells with more DNA are correspondingly larger than cells with less DNA, an observation made across the eukaryote Tree of Life, referred to as the “nucleotypic effect” [33,34,35]. The nucleotypic effect largely depends not on the number of genes but on the amount of non-coding DNA in the form of transposonable elements (TEs), introns and other intergenic regulatory or selectively neutral DNA. More accurately, it is not the DNA content *per se* but the interphase chromosomal volume (ICV), or chromatin state, that determines nuclear and cell size. Nuclear volume thus scales with the amount of space/volume occupied by chromatin [36].

The role non-coding DNA plays in eukaryotes is multifarious, ranging from centromeric and telomeric DNA, which govern mitosis and the number of permissible cell divisions (Hayflick number), to “junk” or “selfish” parasitic DNA [37,38]. Junk DNA—satellite DNA, LINES, SINES, etc.—participates passively in organizing the DNA replication-timing program and the duration of the DNA synthetic phase (S-phase) of the eukaryote cell cycle [39,40]. The so-called junk DNA also participates in the metazoan developmental program and in the cellular response to DNA damage and repair (DDR) [41,42].

Bacteria, in contrast, generally lack a membrane-defined nucleus that compartmentalizes the genome. The genome in bacteria, however, is not a random coil but a membrane-free cellular compartment called the nucleoid. Similar to eukaryotes, cell size is not strictly dependent on the amount of DNA in the genome, but instead scales with the size, or area, of the nucleoid (the ICV in eukaryotes) [43]. Both eukaryotes and prokaryotes, however, increase in cell size with increasing chromosome number (ploidy). As the number of chromosomes increases, so does the cell volume. The largest bacterium described to date, *Thiomargarita magnifica* (9000 micrometers), also has the highest level of polyploidy with half a million copies of a very large genome (11.5 to 12.2 Mb compared to the average bacterial genome size of 4.2 Mb) [44].

### 2.2. Nucleoid Complexity and DNA Replication Fork Rate

While not universal, there is clearly a strong and significant correlation between genome size, polyploidy and cell size/volume across the Tree of Life. Strikingly, the relationship holds under experimental conditions that either increase or decrease DNA content in the cell. One of the most well-established examples is the dependence of cell width (*W*) in *Escherichia coli* and other bacteria on nucleoid complexity (*NC*) or the number of replication points per chromosome: cell width scales with nucleoid complexity in a manner different from but analogous to a nucleotypic effect [23,24].

During thymine-limited growth, DNA replication forks are slower, but DNA content and cell size increase, suggesting that DNA content and cell size are inversely correlated with the replication fork rate ([45,46]; Table 1). A similar effect is observed when cells are treated with hydroxyurea (HU), an inhibitor of ribonucleotide reductase [47]. Under HU, the number of replication forks increases 5- to 10-fold in response to the reduction in fork rate. In eukaryotes, auxiliary origins (dormant origins) fire under the same conditions, likewise increasing the number of replication forks per kilobase [48].

Why and how cell size varies with DNA content remains an unresolved question of both evolutionary and physiological interest. DNA/cell mass homeostasis can explain the long-standing observation that in bacterial cells, initiation of DNA replication occurs at a growth-rate invariant mass at replication initiation, *Mi* [8,49]: the number of replication origins (*oriC*) and cell mass at initiation of DNA replication are independent of cell growth rate. Hence, cell mass per number of origins (number of replication forks) is constant over all growth rates.

**Figure 1:**
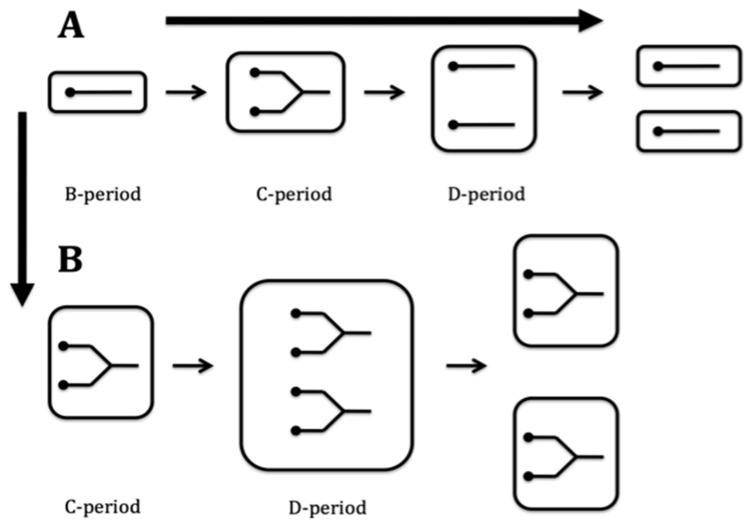
Nucleoid complexity (*NC*) and a nucleotypic effect (*NE*) mediate DNA/cell mass homeostasis under normal and perturbed cell growth conditions. The circles in the stick figures represent DnaA-ATP-licensed origins of replication (*oriC*). The y-shaped (branched) lines represent the number of origins per chromosome (*NC*). The width of the cylinder represents the size of the cell relative to DNA content (*NE*). The top arrow (x-axis) represents cellular productivity (biomass accumulation per cell). The left arrow (y-axis) represents individual cell size (cell-shape homeostasis). (**A**) Nutrient-limited (slow) growth. The successive arrows reflect the transitions between the B-period (Left); C-period (Middle); and D-period (Right). (**B**) Nutrient-unlimited (fast) growth eliminates a B-period.

Cell size in bacteria therefore depends on the number of active replication forks per chromosome (nucleoid complexity: Figure 1). Surprisingly, DNA/cell mass homeostasis is maintained not only under conditions of differing nutrient availability (growth rate) but also under conditions that perturb DNA synthesis either environmentally (DNA replication fork pausing or collapse) or experimentally (inhibiting or slowing replication fork speed: see above).

## 3. DNA Replication Homeostasis

### 3.1. Is There a Conserved (Under All Growth Conditions) Negative Correlation Between DNA Replication Fork Rate and Initiation Frequency (Number of Forks) in Bacterial Cells?

In both prokaryotes and eukaryotes, a negative correlation between replication fork rate and initiation frequency has been consistently observed under a variety of experimental conditions. This suggests a generalized homeostatic mechanism that allows the fork rate to “sense” the number of active replication forks, presumably through competition between active forks for the precursors of DNA synthesis (dNTPs). The negative correlation between fork rate and fork numbers can nevertheless account for how the cell maintains DNA/cell mass homeostasis: the number of replication forks adjusts automatically to replication fork rate such that DNA content increases in parallel to the increase in cell mass during growth. This might suggest that the *Mi* is simply a consequence of the homeostatic mechanisms underlying the equilibrium between the number of forks per chromosome and the corresponding fork rates.

The evidence for a negative correlation between initiation frequency (number of *oriC*/cell cycle) and replication fork rate occurs not only under thymine limitation and hydroxyurea treatment. Advancing initiation timing (reducing *Mi*) also results in a correspondingly longer C-period. In contrast, delaying initiation results in a correspondingly shorter C-period [50,51]. Certain replication elongation mutants extend the C-period, resulting in larger cells at birth and consequently earlier replication timing in the daughter cells, but *Mi* in the daughter cells correlates with *Mi* in the mother cell [47]. Moreover, inhibiting initiation using a *dnaA*ts mutant in an *nrdA101* background at a non-permissive temperature allows for the completion of chromosome replication [52,53]. This observation suggests that inhibition of replication initiation promotes fork processivity in mutants compromised for dNTP synthesis and replication elongation.

Other mutants defective in synthesizing nucleotide precursors have also confirmed the correlation between replication initiation and elongation. A mutant defective in CMP kinase (*cmk*), for example, exhibited similar phenotypes [54]: DNA replication forks slowed, but the frequency of DNA replication initiation increased to compensate exactly for the slower fork rate (a two-fold reduction in the fork rate resulted in a two-fold increase in replication initiation or the number of replication forks). The inverse correlation between dNTP pool turnover (flux) and initiation frequency has been confirmed experimentally by titrating RNR to slow replication fork movement, increase nucleoid complexity and increase cell size [20]. Together, these findings suggest that nucleotide and dNTP pool levels exert a central regulatory role over both cellular and DNA metabolism [55].

These observations are all consistent with the size of the cell scaling with the length of the C-period (and chromosome content). Consequently, cell size should scale inversely with fork rate. According to the Cooper–Helmstetter framework, cell size depends directly on the growth rate:

*M* ∝ *Mi* · 2 ^(C + D)/τ^

where C corresponds to the C-period (constant at 40 min), D corresponds to the D-period (constant at 20 min), and τ corresponds to cell growth rate (variable with nutrient quality). The Cooper–Helmstetter model predicts multi-fork chromosome replication at growth rates of less than 60 min. In contrast to the Cooper–Helmstetter model, if C is variable, then cell mass will depend not only on cell growth rate but also on replication fork rate, and the duration of the C-period will scale with the inverse of the fork rate:

*C* = *L*/υ_f_

where *C* represents the C-period duration, *L* represents genome size, and υ represents the fork rate. Assuming D is constant (though not always), then:

*M* ∝ *Mi* · 2 ^(*L*/υf τ)^

Compare this relationship to *M* ∝ 2 ^(τ(cyc)/τ)^ (where τ_(cyc)_ ∝ 1/υ_f_ under thymine-limiting conditions: Table 2) in Si et al. (2017) [7]. Holding τ constant, it follows that cell size is directly dependent on υ (Table 2); holding υ constant, it follows that cell size is directly dependent on τ, which recovers the Cooper–Helmstetter model and Donachie’s deduction of *Mi*: the number of forks (per growth rate) corresponds to the number of replication origins (*Mi* by definition). In either case, *Mi* would follow naturally from initiation–elongation homeostasis (log*M* ∝ log*Mi* + (*L*/υ_f_ τ)log2, or a straight line with a slope of 1/υ_f_). The above expression predicts not only a relationship between nucleoid complexity (*NC*) and cell width/size but also a nucleotypic effect (*NE*) according to DNA content (see Figure 1), *M* ∝ *L* (cell size increases/scales with DNA content), and the analogy between *NC* and *NE* becomes clearer.

Since nucleoid size (*NS*) scales with cell size (*NS* ∝ *W*), while cell width (*W*) scales with nucleoid complexity (*W* ∝ *NC*), the number of replication forks and cell size, by definition, varies inversely with fork rate: replication initiation is inversely correlated with replication fork rate (elongation). It should be noted, however, that *NS* scaling is independent of DNA replication [43], in agreement with a nucleotypic effect in bacteria: as nucleoid area doubles, cell area doubles. What is the mechanistic basis of this relationship?

Two interpretations of the inverse correlation between the fork rate and initiation frequency have so far been proposed: (1) in eukaryotes, so-called dormant replication origins fire passively before being replicated (and inactivated) when replication forks slow or stall. In bacteria, cell mass continues to accumulate at a constant rate until *Mi* is reached, which then triggers replication initiation; (2) stalled or perturbed replication forks in both prokaryotes and eukaryotes induce the gene coding for the enzyme ribonucleotide reductase [56,57,58], which supplies replication forks and DNA repair systems with essential dNTPs. The latter scenario suggests that the forks themselves control initiation via dNTP synthesis as a necessary, but not sufficient, condition for initiation. Both scenarios, however, are likely to interact synergistically in maintaining DNA/cell mass homeostasis.

**Table 2:** Inverse correlations between replication fork rates and cell mass: cell mass increases as replication fork rate decreases. (For MFA-seq, see [59].)

*Source*	*Figure*	*Growth Condition*	*Mass*	*C-Period* *(min)*	*Fork Rate* *(bp/s)*	*Doubling Time* *(min)*
Si et al. 2017 [7]	Figure 2	Rich	2.5	40	1000	30
Si et al. 2017 [7]	Figure 2	Intermediate	4	60	700	30
Si et al. 2017 [7]	Figure 2	Thymine limited	7.5	100	420	30
Zaritsky 2015 [23]	Figure 1	Strong limitation	8.5	120	350	30
MFA-seq study	Figure 3	Control	_	_	900	_

### 3.2. Do DnaA and RNR Constitute a Homeostatic Pair That Coordinates DNA Replication with the Cell Cycle?

What might be the molecular basis of the inverse correlation between replication initiation and elongation? The DnaA protein is the central regulator of DNA replication initiation. When bound to *oriC* in the ATP form, DnaA-ATP acts to melt the DNA double helix, which permits the loading of the enzymes essential for DNA synthesis and genome duplication (initiation). The enzyme ribonucleotide reductase is the central regulator of DNA replication elongation. As the central regulator, its activity is rate-limiting for replication fork movement and for DNA repair [60].

How these two proteins interact to control DNA metabolism has, to date, attracted little attention, presumably because the interaction is indirect and mediated via dNTP metabolism. Over-expressing RNR, for example, does not directly affect the frequency of replication initiation, while over-expressing DnaA has little direct or mechanistic effect on replication fork rates (C-period), presumably because over-expressing DnaA has a limited effect on either the frequency of initiation or the initiation mass [61]. DnaA, it seems, serves mainly as a licensing factor at *oriC* (similar to the eukaryote Origin Recognition Complex) and not as a timer in initiation control. This, however, remains an open question.

Both the *dnaA* gene and the *nrdAB* gene are cell-cycle-regulated [62,63,64]. DnaA protein synthesis is required for initiation of each division cycle, while RNR synthesis is required for elongation and chromosome duplication. Expression from the *dnaA* gene, however, can be inhibited without affecting initiation, indicating that DnaA is in excess in the cell [65,66]. Both *dnaA* and *nrdAB* are expressed in coordination with initiation, but expression of the two genes apparently oscillates out of phase at slow growth [62]. Although the genes and their products have not been shown to interact directly, low levels of DnaA-ATP have nevertheless been shown to stimulate *nrdAB* expression while high levels act to repress *nrdAB* gene expression [67].

The cell cycle regulation of the two gene products, moreover, appears to be coordinated. While evidence has been found that the DnaA-ATP/DnaA-ADP ratio oscillates during the cell cycle [68], the DnaA protein itself does not oscillate and remains constant, whereas RNR enzyme activity and dNTP pool sizes oscillate in response to the demand for dNTPs during replication. Consequently, dNTP pool levels are low prior to initiation, increase during the C-period and then decrease dramatically when replication terminates, and the cell enters the D-period [69].

This suggests that RNR activity, rather than bulk dNTP pool sizes alone, is rate-limiting for replication forks (see Figure 3 below). RNR is allosterically regulated by dATP (negatively) and depends on the reducing agent NADPH to supply the electrons needed to reduce NDPs to dNDPs [70]. Consequently, high dATP pool levels at the end of the C-period allosterically inactivate RNR [71], indicating that high dNTP pool levels in the absence of active replication forks would inhibit replication re-initiation and DNA synthesis once the chromosome has been fully duplicated. The expected inhibitory effect of dNTP pool size on replication initiation has not been reported in bacteria, but evidence for an effect of high dNTP pool sizes inhibiting the transition to S-phase in eukaryotes has been observed [72].

### 3.3. Does the Bacterial Cell Coordinate dnaA and nrdAB Gene Expression with Cell Growth?

**Figure 2:**
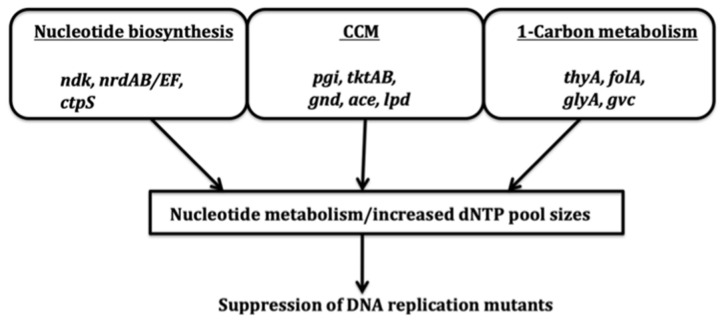
Central Carbon Metabolism (CCM) suppressors of DNA replication mutants converge on nucleotide metabolism to couple the rate of DNA replication (initiation frequency and/or replication fork rate) to the rate of phospholipid/carbon metabolism (ATP supply and redox balance (*aceE*, *lpd*, *ackA*, *pta*, *relA*, *spoT*) [12]). dNTP pool size expansion is one facet of how CCM mutants might suppress *DNA*(ts) mutants. An equally important facet is the increase in dNTP flux from RNR to replication forks when DnaA, or initiation, is either inhibited or restricted, again underscoring a homeostatic relationship between initiation frequency per chromosome (number of forks per chromosome) and the rate of elongation (fork rate). It is proposed here that the coupling suggests a plausible interpretation of how cells maintain DNA/cell mass homeostasis under such a broad range of advantageous and adverse cell growth and DNA replication conditions: the cell membrane (mass) simultaneously grows around, and in proportion to, the growing amount of DNA and vice versa.

Cell size and growth rate depend directly on metabolic rate. Over the past years substantial evidence has accumulated revealing a role of Central Carbon Metabolism (CCM) in the regulation of both replication initiation and elongation [9,10,11,12,13,14,15,16,17] Suppressors of DNA replication mutants fall into four principle categories: ATP supply and redox balance (*aceE*, *lpd*, *ackA*, *pta*, *relA*, and *spoT*), ribose 5-phosphate and energy supply (*pgi*, *tktA/B*, *gnd*, *ace*, and *lpd*), one-carbon/thymidine supply (*thyA*, *folA*, *glyA* and *gvc*), and finally dNTP supply (*ndk*, *nrdAB*/*EF*, and *ctpS*). Importantly, the suppressor mutants do not interact directly with replication factors (DnaA, DnaB, DnaC, DnaE, and DnaQ) but rather indirectly via the metabolites they produce [17]. Hence, directly (via the folate cycle) and indirectly (via the pentose phosphate pathway and the stringent response), the suppressor effect is due to, and converges on, the synthesis and/or flux of dNTPs (Figure 2).

The integration of nucleotide, carbon and energy metabolism guarantees that DNA replication can proceed under a variety of normal and compromising conditions. The homeostatic relationship between replication initiation and elongation also applies to cells that aberrantly initiate DNA replication. In bacteria, for example, the *hda* mutant is defective in hydrolyzing the *oriC*-bound form of DnaA-ATP to the inactive form of DnaA-ADP [73], which is associated with access of SeqA to hemimethylated *oriC* and transient inhibition of initiation (1/3 rd of the cell cycle). Consequently, this mutant over-initiates DNA replication. Over-expressing RNR, however, rescues the cells and restores replication by over-supplying dNTPs [73]. Likewise, over-expressing *nrdAB* rescues the cold-sensitive *dnaAcos* mutant that over-initiates DNA replication at non-permissive temperatures [74]. In eukaryotes, a yeast strain that over-initiates DNA replication is viable if and only if RNR and dNTPs are overproduced [75].

### 3.4. DNA Replication Homeostasis, Mutation and Adaptation

Imbalanced dNTP pools are highly mutagenic [58,76], hence the need for tight regulation of dNTP flux and stringent regulation of RNR and dNTP demand (number of forks, or DnaA-licensed *oriC*s). Tight regulation of the initiation–elongation feedback loop is required to maintain an optimal fork rate and rate of adaptive versus maladaptive mutagenesis. Nucleoside and rNTP pools, however, are 10 to 100X larger than dNTP pool sizes, with the inevitable consequence of NTP incorporation into DNA during DNA synthesis. rNTP incorporation into DNA retards replication fork movement up to 3X or more and promotes mutagenesis and genome instability [77]. This observation would suggest that DNA repair systems (nucleotide and ribonucleotide excision repair) likely accompany, or chaperone (eg. GroEL/ES, DnaK), DNA replication systems [78]. Excision of rNTPs and repair of any related lesions would thus facilitate effective chromosomal duplication, segregation and cell division.

When replication is severely impaired as a result of DNA damage or nutrient starvation, *nrdAB* is induced and RNR activity is up-regulated in both SOS-dependent and -independent manners [79,80,81]. The SOS DNA repair system halts DNA replication until DNA repair factors, including RNR and RecA, can process the DNA lesion [82]. This might indicate that the SOS-independent up-regulation of RNR is in response to retarded rather than fully collapsed DNA replication forks. Although DnaA activity is usually restrained during the SOS response until damage is processed and forks restart, an intriguing possibility, when forks are perturbed and *NC* increases, might be that the cumulating DNA provides substrates for RecA-dependent recombination and repair. DnaA, for example, is induced upon DNA damage [83]. It is not known, however, if *nrdAB* induction and RNR up-regulation respond directly to the increased *NC*, but induction of the *nrdAB* and *dnaA* genes and up-regulation of the RNR enzyme during replication stress is consistent with a homeostatic relationship between fork numbers and fork rates. Increased *NC* and induction of *nrdAB* and *dnaA* during replication stress might therefore represent an adaptation that maintains a stable, optimal mutation/substitution balance over evolutionary time in bacteria.

## 4. Discussion

### 4.1. Summary of Replication Initiation–Elongation Homeostasis

This review has attempted to provide a tentative overview of the coordination between genome duplication and cell growth processes, which, although coupled, are in several ways independent and parallel processes. Duplication of the chromosome, for example, can occur in the absence of cell division, whereas cell division depends on completed DNA replication and sister chromosome segregation (which is prevented via nucleoid occlusion: [84,85,86]. The nucleotypic effect and the analogous nucleoid complexity, generated by initiation/elongation homeostasis, suggest that the number of DNA replication forks and fork rates significantly influence cell size when the DnaA-RNR homeostasis loop is perturbed: fork rates remain strictly inversely correlated with the frequency of replication initiation in all cells examined so far, prokaryote and eukaryote alike, and under all growth conditions including normal growth [87,88].

Perhaps the most direct evidence supporting the hypothesis that DnaA and RNR form a homeostatic pair that sets the *Mi* comes from experiments either over-producing or titrating/inhibiting DnaA or RNR:

(1) DnaA overproduction advances initiation time and thus reduces the initiation mass. Consequently, replication fork rate slows, and the C-period increases to compensate for the perturbed *Mi*.

(2) Conversely, titrating or inhibiting DnaA delays initiation and thus increases the initiation mass in the mother cell. Consequently, replication fork rate increases and the B-period in the daughter cells decreases, again compensating for the perturbed *Mi*.

(3) RNR overproduction increases replication fork rate and decreases the C-period without affecting *Mi*, suggesting a feed-forward relationship between dNTP flux and replication fork rate.

(4) Conversely, titrating or inhibiting RNR decreases replication fork rate and increases the C-period without affecting the D-period.

These observations predict that simultaneously over-producing DnaA and RNR will result in extreme polyploidy and elongated cells, if indeed DnaA and RNR play coordinated roles in maintaining DNA/cell mass homeostasis. Cells defective in cell division (with otherwise normal DNA replication cycles) provide an example of extremely elongated, polyploid cells [89]. A similar phenotype resulting from the simultaneous over-production of DnaA and RNR, however, remains to be demonstrated.

### 4.2. Additional Evidence for the DnaA-RNR Homeostatic Relationship

Other evidence supports the proposal that DnaA-RNR forms a regulatory feedback loop via the coordinated effects they have on the C-period. Inhibiting fork rate by reducing flux through dNTP pools (such as in the *cmk* mutant) under conditions of thymine starvation or by inhibiting RNR with HU results in slower forks and increased nucleoid complexity as well as increased cell volume. Additionally, extra origins slow replication fork rates, but fork rate can be normalized by increasing the available dNTP pool.

Conversely, reducing replication initiation in a *dnaA*(ts) mutant promotes the processivity of replication forks under non-permissive temperature in the *nrdA*101ts mutant. Similarly, reduced initiation efficiency restores viability in temperature-sensitive replisome mutants [90,91]. Thus, inhibiting replication initiation rescues replication forks stressed by dNTP starvation [52,53].

Similar observations have been made in the *dnaA*cos mutant and the *hda* cold-sensitive mutants [73,74]. Over-initiation in these mutants stalls replication forks; up-regulating RNR and expanding dNTP pools, however, rescue retarded fork movement. Other mutants defective in fork processivity reveal a similar increase in nucleoid complexity. Hence, these two parameters, number of forks and fork rate, maintain a correlation between *Mi* in the daughter cells and *Mi* in the mother cell [47]: *Mi* is relatively invariant independently of the mechanism impeding fork rate.

A 2x increase in RNR has also been found to suppress the toxicity of a 10x increase in the β-clamp, the major DNA replication helicase that modulates DNA pol III processivity. This would suggest a regulatory pathway that links *nrdAB* and *dnaAN* gene expression [92]. DNA polymerase is the major determinant of fork rate in E. coli [93] and actively stimulates ATP hydrolysis by DnaA. ATP hydrolysis by DnaA induces the *nrdAB* gene [63]. This observation indicates that dNTP flux must already be sufficient for firing DnaA-licensed *oriC* at the start of the C-period (rather than after DnaA-mediated initiation). DnaA-directed ATP hydrolysis induces *nrdAB* after the eclipse period, thus allowing new replication forks to form and maintaining balanced multi-fork chromosome replication. The DnaA-Hda-DNA pol III-directed hydrolysis of ATP and the concomitant up-regulation of *nrdAB* indicate that dNTP flux must increase to stabilize older replication forks when new replication forks form during the same C-period. Changes in RNR activity/dNTP flux rate thus mediate and stabilize the balance between still active old and newly formed forks as chromosome duplication proceeds toward the terminus.

### 4.3. Mi, (p)ppGpp and the C-Period: A Cell Cycle Clock Under Normal Growth Conditions?

Concentrations of DnaA and ATP in the cell are not only growth-rate-invariant but also remain relatively constant during the cell cycle (via DnaA autoregulation of the *dnaA* promoter, for example). RNR activity, in contrast, is cell-cycle-regulated and oscillates with the C-period in response to dNTP demand (number of replication forks). dNTP synthesis in slow-growing cells is low during the B-period and low during the D-period. Since DnaA is not rate-limiting for the C-period (fork rate) but is rate-limiting for *oriC* licensing following the eclipse period and prior to initiation in the next cell generation, the two factors, DnaA and RNR, must act in concert to license all origins in the cell and to fire them synchronously when dNTP flux becomes permissive. Consequently, new replication forks will not compete with old forks for dNTPs. If this scenario is plausible, then DnaA is necessary to license *oriC*, but dNTPs are necessary to fire the licensed origins.

All of the evidence examined here converges on a homeostatic relationship between the number of replication forks and fork rate: the number of forks and fork rate adjust automatically to each other to maintain a constant net rate of DNA synthesis (constant C-period) in coordination with cell growth rate (DNA replication homeostasis). Replication homeostasis affects cell size (*V*) and cell shape (*W*) homeostasis: an increase/decrease in DNA content (*NC*) correlates with an increase/decrease in cell size (*NE*).

The regulatory involvement of (p)ppGpp illustrates the above principle of tight coordination among these different parameters. (p)ppGpp inhibits replication initiation, and consequently, (p)ppGpp-deficient strains contain multiple replication forks under slow growth conditions [94]. Replication-initiation homeostasis, however, is still maintained: the C-period in (p)ppGpp-deficient cells is extended 3X during slow growth in compensation for the anomalous extra forks, with no significant change in the D-period but an increase of 7X in *Mi* (delayed initiation). Hence, (p)ppGpp-deficient cells are deregulated not only for DNA replication homeostasis but also for cell size homeostasis.

Consequently, (p)ppGpp plays a key role in maintaining a constant growth rate-independent *Mi* during unperturbed growth. (p)ppGpp might therefore serve to integrate these related but different and independent homeostatic mechanisms in order to guarantee that each daughter cell receives an equivalent number of chromosomes from each mother cell. The dynamic coupling between the replication cycle and the cell cycle appears to behave in a synchronized clock-like manner, as originally proposed by Meselson and Stahl. How are these two homeostatic mechanisms synchronized during cell growth?

### 4.4. Coordinating Amino Acid Flux with Nucleotide Flux

**Figure 3:**
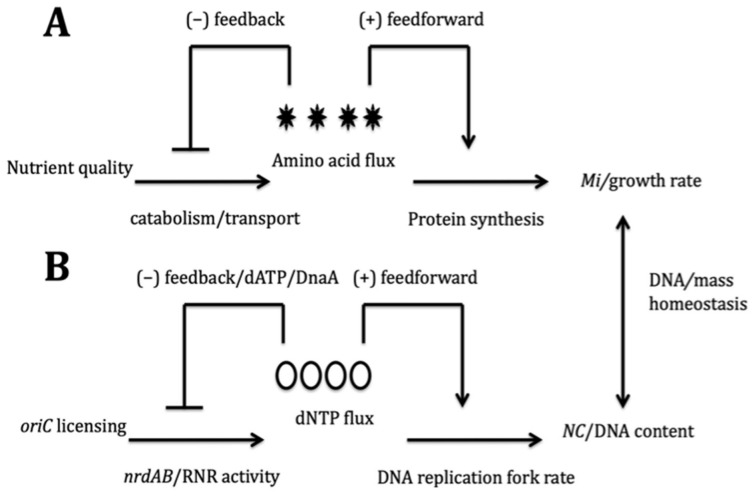
The equilibrium between amino acid synthesis and nucleotide synthesis maintains growth-rate-independent cell size homeostasis and DNA replication homeostasis. (**A**) The nutrient environment (nutrient quality) determines basal metabolic rate (catabolism and transport). The rate of amino acid (aa) synthesis (arrow: aa supply) and protein synthesis (arrow: aa demand) determines aa pool sizes: pool size is minimal when flux is optimal. When pool levels are high, aa pools feedforward (arrow: increase in aa flux) to increase cell growth rate (vertical arrow: protein synthesis). Elevated aa pools result in negative feedback (vertical T-bar) on the aa biosynthesis pathway (adapted from [95]), shifting the equilibrium in favor of the nucleotide biosynthetic pathway (as proposed in [52]). (**B**) It is proposed here that the shift in equilibrium from amino acid to nucleotide biosynthesis occurs, presumably, when DnaA levels are low, which stimulates *nrdAB* expression [67]. As DnaA-ATP progressively saturates the replication origin (*oriC* licensing) and dNTP flux rises, the cell fires DNA synthesis from the origin of replication. Flux through dNTP pools enhances replication fork processivity (vertical arrow: feedforward), which determines the replication fork rate [20]. When flux (dNTP supply) through dNTP pools exceeds fork rate (dNTP demand), the subsequently expanded dATP pools and DnaA-ATP inhibit RNR and the expression of *nrdAB* (vertical T-bar: negative feedback), establishing a sub-saturating equilibrium between dNTP flux and replication fork rate [20]. The two parallel and coordinated (but independent) metabolic pathways (mediated by R5-P availability and (p)ppGpp) thus maintain DNA/mass homeostasis independently of growth rate (vertical double arrow).

The effect of CCM mutants on DNA replication dynamics in both initiation and elongation temperature-sensitive genetic backgrounds might suggest a physiological connection between metabolism (nutrient availability and growth rate) and the initiation–elongation feedback loop. Equilibrium between DnaA activity and RNR activity shifts with growth rate, resulting in the emergence of a B-period at slow growth rates (τ > 60 min). Most, if not all, of the CCM replication *dnaA*(ts) mutant suppressors converge, directly or indirectly, on nucleotide metabolism and dNTP flux (fork rate) (Figure 2 and Figure 3).

**Figure 4:**
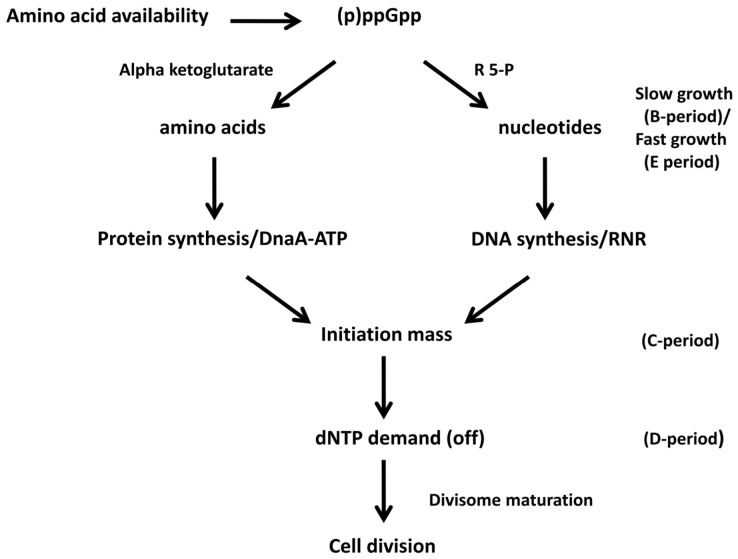
Amino acid flux and nucleotide flux under a slow or a fast growth regime. Experimentally elevating (p)ppGpp levels results in slower cell growth even in nutrient-rich media [101]. (p)ppGpp is also a negative regulator of replication initiation. R 5-P feeds principally into nucleotide biosynthetic pathways. As (p)ppGpp levels decline with increasing nutrient availability, flux through the pentose phosphate pathway (net R 5-P yield) increases nucleotide supply (e.g., dNTPs) and enables replication initiation and elongation. Under a slow growth regime (B-period), negative feedback from elevated amino acid pools reallocates metabolic precursors in favor of nucleotide biosynthetic pathways, activating DNA synthesis at *Mi* (initiation mass). Under a fast growth regime (E-period), new forks form before replication terminates. The DnaA-ATP-Hda-DNA poll III complex de-represses *nrdAB*, allowing dNTP flux/supply to adjust to meet the increased dNTP demand at newly formed replication forks. High pool levels of dNTPs result in negative feedback on dNTP synthesis (dNTP demand “off”), which inhibits replication re-initiation during the D-period. Although hypothetical, the scenario in Figure 4 is consistent with the experimental evidence and other existing hypotheses. The scenario also suggests a corollary to the hypothesis: cell-cycle-dependent dNTP flux might also coordinate replication initiation with cell division, with rising flux in the B-period signaling initiation and declining flux in the D-period signaling cell division.

According to a recent hypothesis, “(p)ppGpp coordinates the dynamic metabolic conversions of nucleotides to amino acids” [55,96,97,98]. It is proposed here that a B-period emerges from a metabolic tradeoff, or re-allocation, between nucleotide metabolism and amino acid metabolism (Figure 3 and Figure 4). The tradeoff might reflect (p)ppGpp levels, which vary inversely with growth rate [99,100]. Under nutrient/amino acid scarcity, low nucleotide pools and slow rates of one-carbon and amino acid metabolism limit growth and delay replication initiation until sufficient biomass has accumulated (*Mi*) to shift metabolic equilibrium in favor of nucleotide synthesis and replication initiation–elongation: DnaA-ATP-licensed origins fire only when dNTP flux rate reaches a permissive threshold (Figure 4). This, however, remains to be demonstrated experimentally, for example, by inhibiting RNR while over-producing DnaA during the B-period in order to probe a corresponding delay in initiation timing.

## 5. Conclusions

Many other questions emerge from the evidence establishing a homeostatic interaction between replication initiation and elongation, as well as from the preliminary evidence that DnaA and RNR constitute a homeostatic pair. Consistent with high levels of the β-clamp helicase, high levels of DnaA also inhibit cell growth [102], whereas high levels of DnaA inhibit, at the same time, *nrdAB* expression and dNTP flux [67]. According to the model proposed here, the DnaA-RNR feedback loop would prevent origins from firing due to insufficient dNTP flux. Conversely, in yeast, it has been shown that larger-than-normal dNTP pools inhibit replication initiation and entry into S-phase, but a similar phenomenon has yet to be shown in bacteria: do high dNTP pools likewise inhibit initiation in bacteria? Another related question concerns (p)ppGpp regulation of replication initiation in the slow growth regime. In cells with defective (p)ppGpp, replication initiation (o/t ratio) increases 2x while the C-period concomitantly increases 3X [102]. Does this reflect competition for ribose 5-phosphate between amino acid and nucleotide biosynthesis (Figure 4)? Competition for ribose 5-phosphate might lead to premature and elevated nucleotide biosynthesis, and therefore to the aberrant, untimely firing of replication origins.

Additionally, high dNTP pool levels due to low dNTP demand and flux at the end of DNA synthesis might add another layer to the negative regulation of premature origin firing in *E. coli* during the D-period. Another question concerns the ATP/ADP status of DnaA in *oriC*^allADP^-modified cells, in which DnaA-ADP binds the modified origin with the same efficiency as DnaA-ATP, but *oriC*^allADP^ cells display a hyper-initiation phenotype (similar to *hda*/*dnaA*cos) [103]. Is *nrdAB* expression, which is regulated by DnaA-ATP, altered or normal, given that DnaA-ADP/*oriC*^allADP^ suppresses the lethality of DnaA mutants defective in ATP binding? This raises a more general question: do most suppressors of *DNA*(ts) mutants operate through a common pathway that converges, directly or indirectly, on nucleotide biosynthesis and dNTP flux? The emerging evidence that DnaA and RNR form a complex, incompletely understood homeostatic pair might have the yet-unrealized potential to explain, at least in part, the long-standing mystery of the multiple mechanisms that determine the initiation mass in bacteria.

## Figures and Tables

**Table 1:** *E. coli* cell dimensions at different growth rates and corresponding nucleoid complexities (data from Zaritsky 2015 [23]; see also Howard 2024 [24]). The table shows a 3× decrease in *τ* (from 51.53 to 17.10) and an approximately 2× increase in *NC* (from 1.366 to 2.776).

*τ (min)*	*W (μm)*	*n = C/τ*	*NC = o/t*	*W/NC*
51.53	0.55	0.857	1.366	0.4026
50.85	0.56	0.865	1.370	0.4088
37.70	0.64	1.167	1.540	0.4156
30.15	0.71	1.459	1.730	0.4100
26.65	0.72	1.651	1.870	0.3851
22.50	0.85	1.956	2.124	0.4002
17.10	1.04	2.573	2.776	0.3746

## Data Availability

No new data were created or analyzed in this study.
